# Impact of dietary pomegranate peel biochar on performance and economics of Nile tilapia (*Oreochromis niloticus*)

**DOI:** 10.1038/s41598-025-28173-5

**Published:** 2025-12-01

**Authors:** Shimaa Tarek Mohamed Kasseh, Ibrahim Mohamed Abaza, Adel Hassan Saad, Mayada M. H. Khalil, Samia Fawzy, Essam Menshawy

**Affiliations:** 1 Department of Animal and Fish Production, Faculty of Desert and Environmental Agriculture, Matrouh University, Matrouh, Egypt; 2 Nutrition and Clinical Nutrition Department, Faculty of Veterinary Medicine, Matrouh University, Matrouh, 51744 Egypt; 3 Department of Animal Wealth Development, Faculty of Veterinary Medicine, Kafrelsheikh University, Kafrelsheikh, Egypt; 4 Department of Animal and Fish production - Faculty of Agriculture, Alexandria University, Alexandria, Egypt

**Keywords:** Antioxidant activity, Food waste, Growth, Nile tilapia, Pomegranate peel, Biochemistry, Physiology

## Abstract

This study sought to ascertain the actual effects of different dietary levels of pomegranate peel biochar on the performance, antioxidant capacity, intestinal and liver histology, and expression of growth, antioxidant, and immune-related genes, as well as the economics of Nile tilapia (*Oreochromis niloticus*). One hundred and twenty fingerlings (3.15 ± 0.15 g/fish) were randomly distributed into 4 triplicate groups (10 fish/replicate). The pomegranate peel was added to the fish diet at 1, 3, and 5 g/kg diet as Bp1%, Bp3%, and Bp5%. They were given to Nile tilapia for 60 days. Fish fed a diet supplemented with pomegranate peel displayed higher growth (*P* < 0.05) and antioxidants with normal histology of the liver and spleen. Additionally, the intestinal villi length and goblet cells increased (*P* < 0.05) with increasing levels of biochar with the overexpression of the growth, antioxidant, and immune-related genes. The return parameters also showed the same trend, as the highest total return, net profit and rate of return on investment were recorded for fish-fed pomegranate peel-supplemented diets. Thus, pomegranate peel biochar could be considered a promising cost-effective supplement with the potential to improve Nile tilapia’s growth performance, antioxidant status, and economic feasibility.

## Introduction

The fastest-growing food production industry in the world, aquaculture is recognized as an essential component of numerous initiatives aimed at reducing poverty worldwide^1^. One of the most widely consumed foods and an important source of animal protein is fish and seafood. After carp, Nile tilapia is the second most farmed freshwater fish^2–4^. Egypt produces 17.4% of the world’s tilapia, making it the most farmed freshwater fish and the only African country in the top ten tilapia production rankings^2,5^. Therefore, developing tilapia farming is an absolute necessity and a primary goal for various fields of scientific research.

In order to maximize fish productivity and welfare, attention should be paid to the oxidative status of fish because it is directly related to the health, growth, and quality of fish^6^. In this context, natural antioxidants have received considerable attention recently, as they are safer and more effective than synthetic antioxidants. Moreover, conventional antioxidants are widely accepted by the consumers all over the world owing to their safety along with health benefits^7^. To find cheaper, safer, and more efficient natural ingredient substitutes, a lot of research has been done on herbs, vegetables, and other food plants^8,9^. One of the potential components is the pomegranate (*Punica granatum*), a plant in the *Punicaceae* family. Pomegranate oil, juice, and peel all have a lot of antioxidants and strong anti-inflammatory properties^10,11^. They are therefore widely used in medicine, cosmetics, and as a possibly beneficial nutritional addition to animal feed^12^. Pomegranate juice is among the most widely consumed items manufactured from pomegranate fruit^13^. Juice production generates a lot of waste and byproducts; thus, recycling them all is essential. Ali et al.^14^ state that one of the main food industry wastes is pomegranate peel, which makes up over half of the fruit. Pomegranate peels have a higher total phenolic content and antioxidant activity than the fruit’s pulp, blossom, leaf, and seed, despite being an agricultural waste^11^. In the food processing and animal feed industries, these peels could therefore be a special source of bioactive compounds^15,16^. Research has demonstrated that the addition of herbal extracts to the diet of Nile tilapia can enhance growth rate, feed efficiency, immunological function, and oxidation resistance^9,17–19^. Likewise, previous research has indicated the beneficial effects of pomegranate usage in the aquaculture industry. Sarfraz et al.^20^ revealed that the growth performance, body composition, feed efficiency, antioxidant status, in addition to the immunological and hematological parameters of fish, were efficiently improved after consumption of a pomegranate-contained diet. Pomegranate seeds were found to act as appetizers and consequently, growth promoters for fish due to their contents of phenolics and tannin^21^. The growth of rainbow trout (*Oncorhynchus mykiss*) was enhanced, while AST and ALT were reduced by dietary supplementation of pomegranate seed oil^10^. Besides, the incorporation of pomegranate peel in the diet of Nile tilapia was suggested to improve their growth performance^9^. Furthermore, pomegranate feeding was reported to increase the activities of lysozyme, SOD and CAT in *O. niloticus*^19^, in addition to protecting fish against *Aeromonas hydrophila* and metal toxicity^22^. However, pomegranate peel is rarely used as a feed component in fish feed, nevertheless. Consequently, it is critical to shed additional insight into how this substance affects fish performance. Moreover, data on the economic analysis of using pomegranate peel as an additive in fish feed are lacking. Therefore, the purpose of this study was to examine how pomegranate peel biochar meal affected the Nile tilapia’s antioxidant activity, growth performance, body composition, and histological examination as a direct result. Additionally, the expression of the growth, antioxidant, and immune-related genes in the liver tissue.

## Materials and methods

### Experimental design

The present study was carried out at Alexandria University in Egypt, at the Poultry and Fish Production Department of the Fish Research Facility of Agriculture. A local fish farm from the Governorate of ALEX, Egypt, supplied the Nile tilapia. After arrival, the fish were kept in glass aquaria for 14 days before the beginning of the feeding trail for acclimatization. During this period, fish were fed with a commercial tilapia diet (30% CP). A total of 120 fish, weighing an average of 3.15 ± 0.15 g/fish, were divided at random into four triplicate groups (30 fish/group, and 10 fish/replicate) among 12 glass aquariums (each holding 80 L) filled with fresh water that was aerated and devoid of chlorine. Aeration was supplied using an air blower. The initial feeding rate was adjusted to 5% of the fish body weight, then it was reduced every 2 weeks according to the biomass in each aquarium. Every two weeks, the fish were weighed individually to track their growth and adjust the feeding rate. Three feedings were given to the experimental fish at nine, eleven, and one o’clock in the morning. A day prior to the final weighing, feeding was stopped. About 25–30% of the water in each aquarium was removed and replaced with clean de-chlorinated water two to three times weekly. Digital YSI (**APHA**,** 1995**) was used daily to detect the temperature and dissolved oxygen in each tank. pH and ammonia levels were checked twice a week. Measurements of water quality were kept within the typical range for tilapia rearing, and the average values were (± SD): The water’s temperature was 26 ± 2.5 °C, its dissolved oxygen content was 6.7 ± 0.3 mg/l, its total ammonia content was 0.22 ± 0.1 mg/l, and its pH was 7.2 ± 0.1.

This study was approved by Faculty of Agriculture, Alexandria University, Egypt (Serial No. 0108541). We obtained informed consent through an email from the owner to use the fish in our study. All methods were carried out in accordance with relevant guidelines and regulations. Also, the authors confirm that the study was carried out in compliance with the ARRIVE guidelines.

### Pomegranate peel preparation and experimental diets manufacturing

The Agriculture Research Center (Giza, Egypt) provided the pomegranate fruits (*Punica granatum*). The raw materials were Pomegranate wastes (PW), which accounted for roughly 54% of the fruit and were the waste components of pomegranate after juice extraction. The PW was washed several times with distilled water to remove extraneous materials such as dirt, sand, and other impurities. PW was converted into biochar through slow pyrolysis at 300 °C for 20 min in the absence of an oxygen environment in a muffle furnace^23^. The feedstock was placed in a ceramic vessel, following the procedures described by^24^. The resulting biochar was referred to as pomegranate waste biochar (PWB). After the carbonization, the sample was kept for cooling to ambient temperature, after which the percentage yield of biochar was calculated. Then the biochar sample was ground and sieved to 0.5 mm sized particles using a plastic sieve. After several rinses with deionized (DI) water to remove impurities such as ash, the RHB sample was dried at 80 °C for further tests.

The chemical composition of the prepared meals is displayed in Table 1. Every other ingredient used to prepare the diet was bought commercially. Three diets (designated Bp1%, Bp3%, and Bp5%) were supplemented with dried pomegranate peel at 1, 3, and 5 g/kg diet. A control meal comprising 4000 kcal/kg and 30% crude protein was created to satisfy the nutritional requirements of the fish. Water and vegetable oil were added after the ingredients had been well combined and weighed. The pellets were made in an Egyptian J. Nutrition and Feeds (2023) pelletizer. Before being used, the pellets were stored at 5 °C in plastic bags after being sun-dried. Every therapy was given to each replicate six days a week.

**Table 1:** Feed ingredients (%) and proximate chemical analysis (%) on DM basis of the experimental diet used in the first experiment.

Item	Experimental groups			
	Control	Bp1%	Bp3%	Bp5%
Feed ingredients (g 100 g-1)				
Fish meal	17	17	17	17
Corn gluten	10	10	10	10
Soybean meal	20	20	20	20
Yellow corn	20	20	20	20
Wheat bran	10	10	10	10
Rice bran	15	15	15	15
Premix	2	2	2	2
Plant oil	6	6	6	6
Biochar of pomegranate peel	-	1	3	5
Proximate chemical analysis (%) on DM basis				
Dry matter (DM)	97.2	97.2	97.2	97.2
Crud protein (CP)	34.43	34.43	34.43	34.43
Ether extract	9.81	9.81	9.81	9.81
Ash	17.20	17.20	17.20	17.20
Crude fiber	2,22	2,22	2,22	2,22
Nitrogen free extract (NFE)	36.34	36.34	36.34	36.34

### Growth performance and sample analysis

At the end of the experiment, the feed was stopped for 24 h. prior to sampling. All fish in each aquarium were individually weighed and counted to calculate the following:

$$\text{Weight}\:\text{gain}\:\text{(g)}=\:\text{FBW}\:\text{(g)}-\:\text{IBW}\:\text{g}$$

$$\:\text{Weight}\:\text{gain}\:\text{in}\:\text{percent}\:\left(\text{WG}\:{\%}\right)\text{=}\frac{\left[\text{FBW}\:\left(\text{g}\right)-\:\text{IBW}\:\left(\text{g}\right)\right]\times\text{100}}{\text{days}\:\text{of}\:\text{experiment}}$$

$$\:\text{Specific}\:\text{growth}\:\text{rate}\:\left(\text{SGR}{\%}\right)=\frac{\text{[ln (FBW (g))- ln (IBW (g))]}\times100}{\text{days}\text{}\text{of experiment}}$$

$$\:\text{FCR}=\frac{\text{Feed Intake (g)}}{\text{Weight gain}\text{(g)}}$$.

After 8 weeks (end of the experiment) and before sampling, fish were anesthetized with 25 mg/L of tricaine methane sulphonate (MS-222). Blood was collected from the caudal vein without anticoagulant to separate serum (9 fish/group, three/aquarium tank). The serum samples were separated by centrifugation of coagulated blood at 3000 rpm for 15 min. The separated serum was kept at −20 °C until use to determine the level of total protein, albumin, creatinine, urea, malondialdehyde (MDA), and total antioxidant capacity (TAC) and activity of ALT, AST, and catalase (CAT). All serum parameters were determined using commercial kits (Diagnostic and Research Reagents from the company of Bio-Diagnostic). While globulin was calculated by mathematical subtraction of the albumin value from that of the total protein.

Also, at the end of the experiment, experimental diets and 9 fish/group (3 fish/aquarium tank) were sampled and used for diet and body, respectively, proximate composition analysis^25^. The whole fish bodies were used to determine chemical composition. The moisture content was determined by drying samples at 105 °C for 24 h in a dry oven. The Kjeldahl method was used to assess the crude protein content, while the Soxhlet extraction method was used for evaluating the lipids, and ash was determined using a furnace muffler at 600 °C for 2 h.

### Histopathology

Following the collection of blood, the fish were put to euthanize in a 50 mL/L clove oil bath (Barijesans Co., Kashan, Iran) and carefully dissected in order to get tissue samples. Fish from various groups had their livers and intestines sampled for histological analysis. Following the collection of liver and intestinal tissue from various groups, it was preserved in 10% neutral buffered formalin. Following dehydration and clearing, the tissues were sectioned at a thickness of 5 μm and embedded in paraffin. Hematoxylin and eosin staining was applied to the serial slices^26^. The histomorphometric analysis was performed using ImageJ analysis software (National Institutes of Health, MD, USA), whereas the intestinal villi length, width, and the inter-villi space were measured by ImageJ analysis software and expressed as µm. The program was free on the web under https://imagej.nih.gov/ij/download.html.

## Gene expression

### The relative mRNA expression

For the relative mRNA expression, the collected liver samples (9 fish/group (3 fish/aquarium tank)) were immediately frozen in liquid nitrogen and stored at −80 °C. Then, total RNA was extracted using Trizol following the guidelines of the manufacturer **(**iNtRON Biotechnology, Inc., Korea**).** The quality of RNA was verified by agarose gel electrophoresis, while its concentration was determined by nanodrop. The mRNA was reverse transcribed according to the manufacturer’s procedures **(**2X RT Mix, Applied Biotechnology, Egypt**)**.

Genes-specific primer pairs were used for the assessment of the expression of some antioxidant genes (*CAT* and *GPX*), innate immune response genes *(LZM* and *C3*) and growth-related genes (*GHR1* and *IGF1*) (listed in the primers in Table 2). The gene amplification was done using the quantitative Real-Time PCR (PikoReal, Thermoscientific, TCR0024), using the 2x -Lo-Rox- SYBR green kits (Applied Biotechnology, Egypt). The amplification conditions and the reaction mix were done according to Abdo et al.^27^. The annealing temperature was at 60 °C/30 sec. CT values for each sample were estimated and combined in fold change (2^−ΔΔCT^) calculation; the data were normalized against the house keeping gene (Ef-1α) and the control group (non-supplemented) according to Livak and Schmittgen^28^.

**Table 2:** Primer sequence used in real time PCR.

Gene	Primer	Accession No	References
*Ef1*	F: TCAACGCTCAGGTCATCATC R: ACGGTCGATCTTCTCAACCA	XM_003458541	^29^
*CAT*	F: CCCAGCTCTTCATCCAGAAAC R: GCCTCCGCATTGTACTTCTT	JF801726.1	^30^
*GPX*	F: CCAAGAGAACTGCAAGAACGA R: CAGGACACGTCATTCCTACAC	DQ355022	
*LZM*	F: AAGGGAAGCAGCAGCAGTTGTG R: CGTCCATGCCGTTAGCCTTGAG	XM_003460550.2	^31^
*C3*	F: GGTGTGGATGCACCTGAGAA R: GGGAAATCGGTACTTGGCCT	XM_013274267.2	
*GHR1*	F: CAGACTTCTACGCTCAGGTC R: CTGGATTCTGAGTTGCTGTC	MW509678.1	^32^
*IGF1*	F: GTTTGTCTGTGGAGAGCGAGG R: GAAGCAGCACTCGTCCACG	NM_001279503.1	

*Ef-1α*: elongation factor-1α (ef-1α); *CAT*: Catalase; *GPX*: glutathione peroxidase; *GHR1*: growth hormone receptors 1; *IGF-1*: insulin-like growth factor 1; *LZM*: lysozyme gene; *C3*: complement 3.

### Calculation of economic indices

Economic indices were calculated using cost and return parameters. The costs included total variable cost (TVC), total fixed cost (TFC), and total cost (TC). Feed cost was considered by multiplying the FI by the cost of 1 kg of feed (13 EGP/kg feed). The cost of pomegranate peel biochar (0.1, 0.3, and 0.5 EGP/kg for the 1%, 3%, and 5% treatments, respectively) was added to the feed costs of the experimental groups to represent the variable costs. Other costs, including the price of fries, equipment, disinfectant, veterinary supervision, labor, drugs, and vaccines, were reflected in fixed expenses as they are identical and equal (4 EGP). The total costs are the sum of fixed and variable costs. Total return obtained from selling fish. The net profit (NP) was calculated as the difference between the total return and total costs. Rate of return on investment obtained from the equation: RRI = NP/TC*100, while profitability index (PI) = NP/TR. The economic parameters were calculated according to Mahboub et al.^33^. The costs and returns calculations were based on the market prices that prevailed during the period of the study.

### Analytical statistic

The minimum sample size required for a research study was ascertained by power analysis prior to the experiment’s commencement. Additionally, in order to demonstrate homoscedasticity and normality, the data were subjected to the Shapiro-Wilk and Levene tests for normal distribution. The SPSS 19 program (SPSS Inc., IL, USA) was used to do a one-way ANOVA. The Duncan’s innovative multiple range test^34^ was used to compare the means (means ± SD, standard deviation) of the various treatments, where treatment differences were considered significant (*P* < 0.05).

## Results

### Growth performance

Table 3 shows the growth performance of *O. niloticus* as affected by pomegranate peel biochar additives. The final body weight and weight gain showed significant (*P* ≤ 0.05) increases in the Bp3% and Bp5% groups with increased biochar concentration when compared with the control and Bp% groups. Meanwhile, FCR showed a significant (*P* ≤ 0.05) decrease in the Bp1%, Bp3%, and Bp5% groups when compared with the control group. Moreover, the ADG and FI increased significantly (*P* ≤ 0.05) in the Bp5% group when compared with the control and Bp1% groups.

**Table 3:** Effect of different doses of biochar of pomegranate peel on growth parameters of Nile Tilapia for 60 days.

Parameters Groups	Intial body weight	Final body weight	Weight gain	Average daily gain	Feed intake	FCR
Control	3.17 ± 0.09	11.82 ± 0.10 ^c^	8.72 ± 0.16 ^c^	0.14 ± 0.00 ^c^	17.29 ± 0.18 ^c^	2.05 ± 0.00 ^a^
Bp1%	3.13 ± 0.08	12.14 ± 0.02 ^c^	9.01 ± 0.09 ^c^	0.15 ± 0.00 ^bc^	17.81 ± 0.12 ^b^	1.92 ± 0.02 ^b^
Bp3%	3.10 ± 0.06	12.73 ± 0.15^b^	9.53 ± 0.14 ^b^	0.16 ± 0.00 ^ab^	18.12 ± 0.06 ^ab^	1.92 ± 0.04 ^b^
Bp5%	3.20 ± 0.06	13.18 ± 0.15^a^	10.02 ± 0.03 ^a^	0.17 ± 0.00 ^a^	18.29 ± 0.06 ^a^	1.83 ± 0.04 ^b^

Values are means ± standard error. Mean values with different letters at the same column significantly differ at (*P* ≤ 0.05). The absence of letter means that there were no significant differences between treatments.

### Proximate analysis of the fish body

Table 4 shows the chemical composition of *O. niloticus* as affected by pomegranate peel biochar additives. The moisture% and ash showed a significant (*P* ≤ 0.05) increase in the pomegranate peel biochar groups with increased biochar concentration when compared with the control group. Meanwhile, the crude protein increased significantly (*P* ≤ 0.05) in the Bp1% group when compared with the other groups. Additionally, ether extract significantly (*P* ≤ 0.05) increased in the control group when compared with the biochar groups.

**Table 4:** Effect of different doses of Biochar of pomegranate Peel on chemical composition of nile tilapia body for 60 days.

Parameters Groups	Moisture	Crude protein	Ether extract	Ash
Control	71.05 ± 0.01^c^	53.61 ± 0.39 ^b^	26.98 ± 0.48 ^a^	20.71 ± 0.17 ^c^
Bp1%	72.77 ± 0.15 ^b^	54.31 ± 0.06 ^a^	25.33 ± 0.01 ^bc^	21.09 ± 0.01^b^
Bp3%	75.67 ± 0.01 ^a^	50.85 ± 0.03 ^c^	25.56 ± 0.02 ^b^	21.71 ± 0.05 ^a^
Bp5%	75.55 ± 0.06 ^a^	49.68 ± 0.01 ^d^	24.6 ± 0.06 ^c^	21.95 ± 0.01 ^a^

Values are means ± standard error. Mean values with different letters at the same column significantly differ at (*P* ≤ 0.05). The absence of letter means that there were no significant differences between treatments.

### Biochemical parameters

Table 5 shows the biochemical parameters of *O. niloticus* as affected by pomegranate peel biochar additives. The activity of AST and level of creatinine showed a significant (*P* ≤ 0.05) decrease in the pomegranate peel biochar groups with increased biochar concentration when compared with the control group. Meanwhile, the ALT activity and urea level decreased significantly (*P* ≤ 0.05) in the Bp3% and Bp5% groups when compared with the Bp1% and control groups. Additionally, total protein and albumin significantly (*P* ≤ 0.05) increased in the biochar groups when compared with the control group. Meanwhile, the globulin levels increased in the Bp3% group when compared with the other groups.

**Table 5:** Effect of different doses of Biochar of pomegranate Peel on serum biochemistry of nile tilapia for 60 days.

Parameters Groups	AST (U/L)	ALT (U/L)	Total protein (g/dl)	Albumin (g/dl)	Globulin (g/dl)	Creatinine (mg/dl)	Urea (mg/dl)
Control	63.5 ± 0.87 ^a^	27.5 ± 1.44 ^a^	3.80 ± 0.03 ^d^	1.27 ± 0.01 ^c^	2.52 ± 0.01 ^b^	1.03 ± 0.01 ^a^	5.14 ± 0.07 ^a^
Bp1%	52.5 ± 0.58 ^b^	26.5 ± 0.29 ^a^	3.92 ± 0.01 ^c^	1.39 ± 0.02 ^b^	2.54 ± 0.03 ^b^	0.87 ± 0.01 ^b^	5.42 ± 0.27 ^a^
Bp3%	52.5 ± 0.87 ^b^	23 ± 0.58 ^b^	4.10 ± 0.03 ^b^	1.39 ± 0.01 ^b^	2.72 ± 0.02 ^a^	0.9 ± 0.02 ^b^	4.5 ± 0.13 ^b^
Bp5%	41 ± 0.58 ^c^	18.5 ± 0.87 ^c^	4.46 ± 0.02 ^a^	1.95 ± 0.03 ^a^	2.51 ± 0.01 ^b^	0.62 ± 0.01 ^c^	3.09 ± 0.02 ^c^

Values are means ± standard error. Mean values with different letters at the same column significantly differ at (*P* ≤ 0.05). The absence of letter means that there were no significant differences between treatments.

### Antioxidant

Table 6 shows the serum antioxidant parameters of *O. niloticus* as affected by pomegranate peel biochar additives. The activity of CAT and level of TAC showed a significant (*P* ≤ 0.05) increase in the pomegranate peel biochar groups with increased biochar concentration. Meanwhile, the MDA level decreased significantly (*P* ≤ 0.05) in the Bp5% group when compared with the Bp1%, Bp3%, and control groups.

**Table 6:** Effect of different doses of Biochar of pomegranate Peel on antioxidant of nile tilapia for 60 days.

Parameters Groups	MDA (nmol/ml)	CAT (U/ml)	TAC (ng/ml)
**Control**	4.70 ± 0.32 ^a^	2.38 ± 0.13 ^d^	0.82 ± 0.04 ^c^
**Bp1%**	4.68 ± 0.04 ^a^	3.10 ± 0.03 ^c^	1.11 ± 0.01 ^b^
**Bp3%**	4.28 ± 0.17 ^a^	3.36 ± 0.08 ^b^	1.08 ± 0.06 ^b^
**Bp5%**	3. 17 ± 0.01 ^b^	5.60 ± 0.03 ^a^	1.5 ± 0.01 ^a^

Values are means ± standard error. Mean values with different letters at the same column significantly differ at (*P* ≤ 0.05). The absence of letter means that there were no significant differences between treatments.

### Histology

**Fig. 1:**
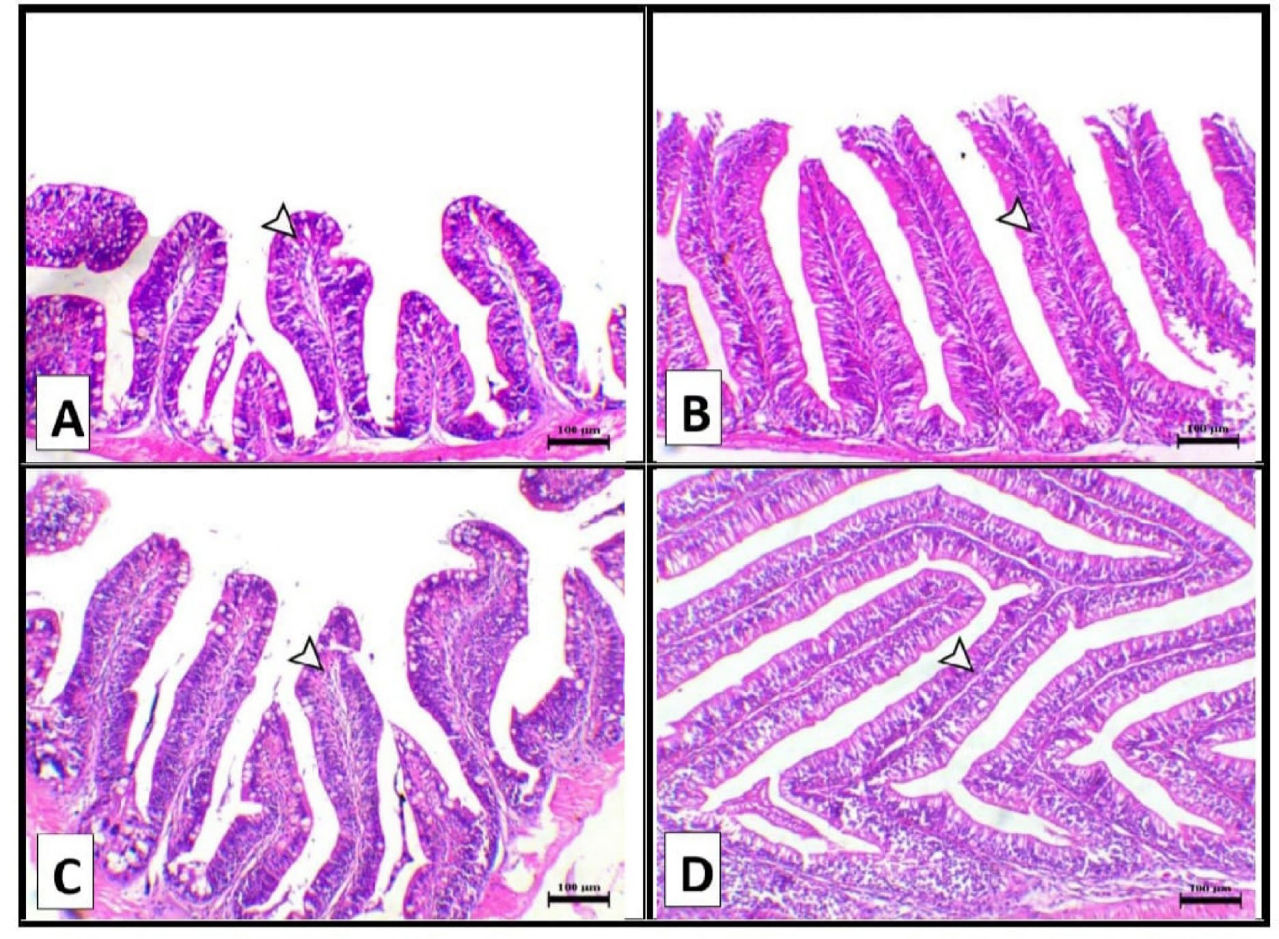
Showed the middle portion of the intestine of the different groups. (**A**) Intestine (middle portion) of fish supplemented with control diet showing normal villi lined with pseudostratified epithelium with goblet cells (arrowhead). (**B**) Intestine (middle portion) of fish supplemented with biochar of Pomegranate peel 1gm/100 gm diet showing mild increase of intestinal villi length (arrowhead). (**C**) Intestine (middle portion) of fish supplemented with biochar of Pomegranate peel 3gm/100 gm diet showing mild increase of intestinal villi length (arrowhead). (**D**) Intestine (middle portion) of fish supplemented with biochar of Pomegranate peel 5gm/100 gm diet showing marked increase of intestinal villi length (arrowhead), H&E, X100, bar = 100 μm.

**Fig. 2:**
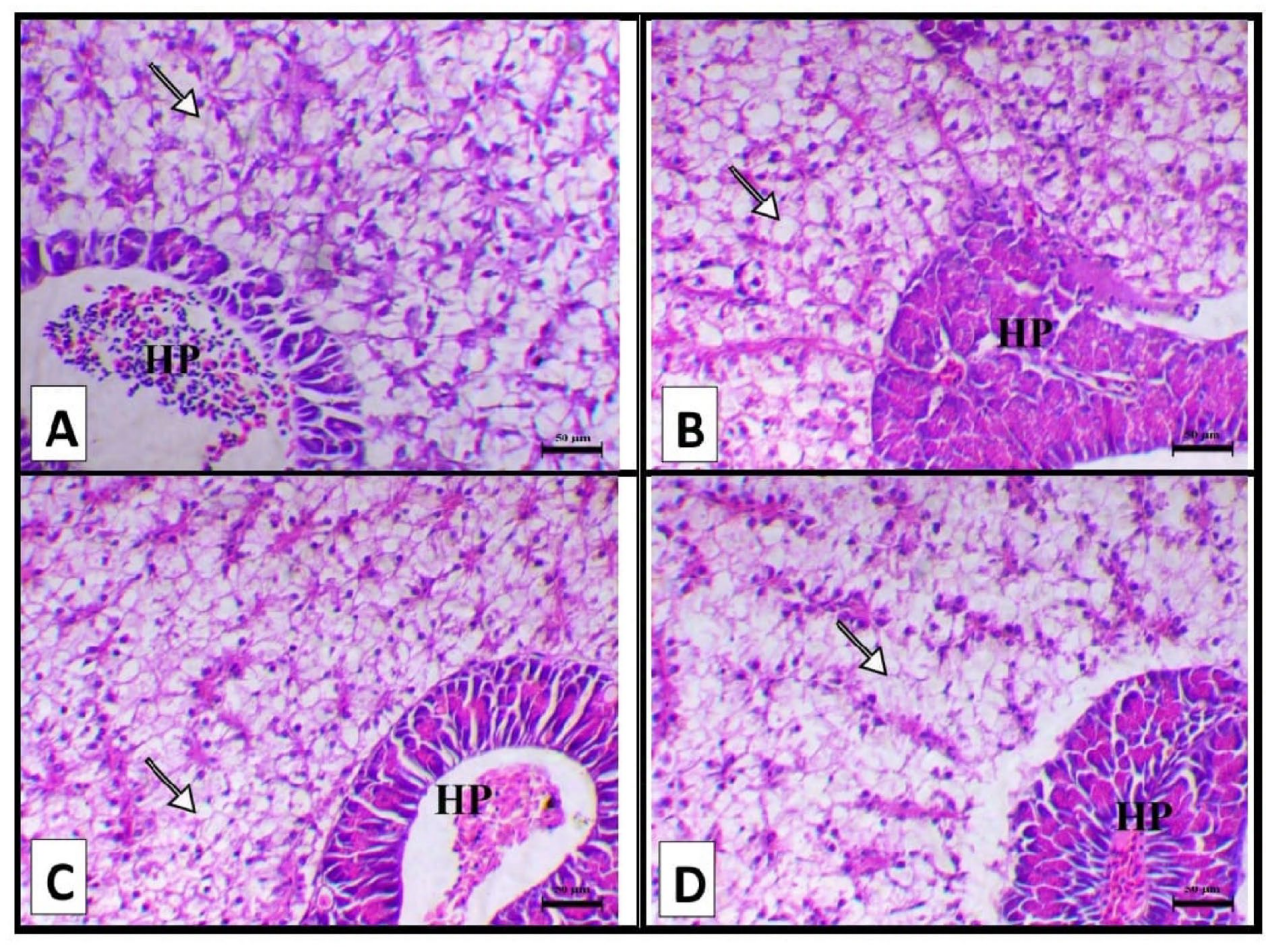
Showed the liver of the different groups. (**A**) Liver of fish supplemented with control diet showing normal hepatic and pancreatic portions (HP) with marked hepatic vacuolation (arrow). (**B**) Liver of fish supplemented with biochar of Pomegranate peel 1gm/100 gm diet showing normal hepatic and pancreatic portions (HP) (arrow indicates decrease hepatic vacuolation). (**C**) Liver of fish of fish supplemented with biochar of Pomegranate peel 3gm/100 gm diet showing normal hepatic and pancreatic portions (HP) (arrow indicates decrease hepatic vacuolation). (**D**) Liver of fish supplemented with biochar of Pomegranate peel 5gm/100 gm diet showing normal hepatic and pancreatic portions (HP) (arrow indicates decrease hepatic vacuolation), H&E, X100, bar = 50 μm.

**Fig. 3:**
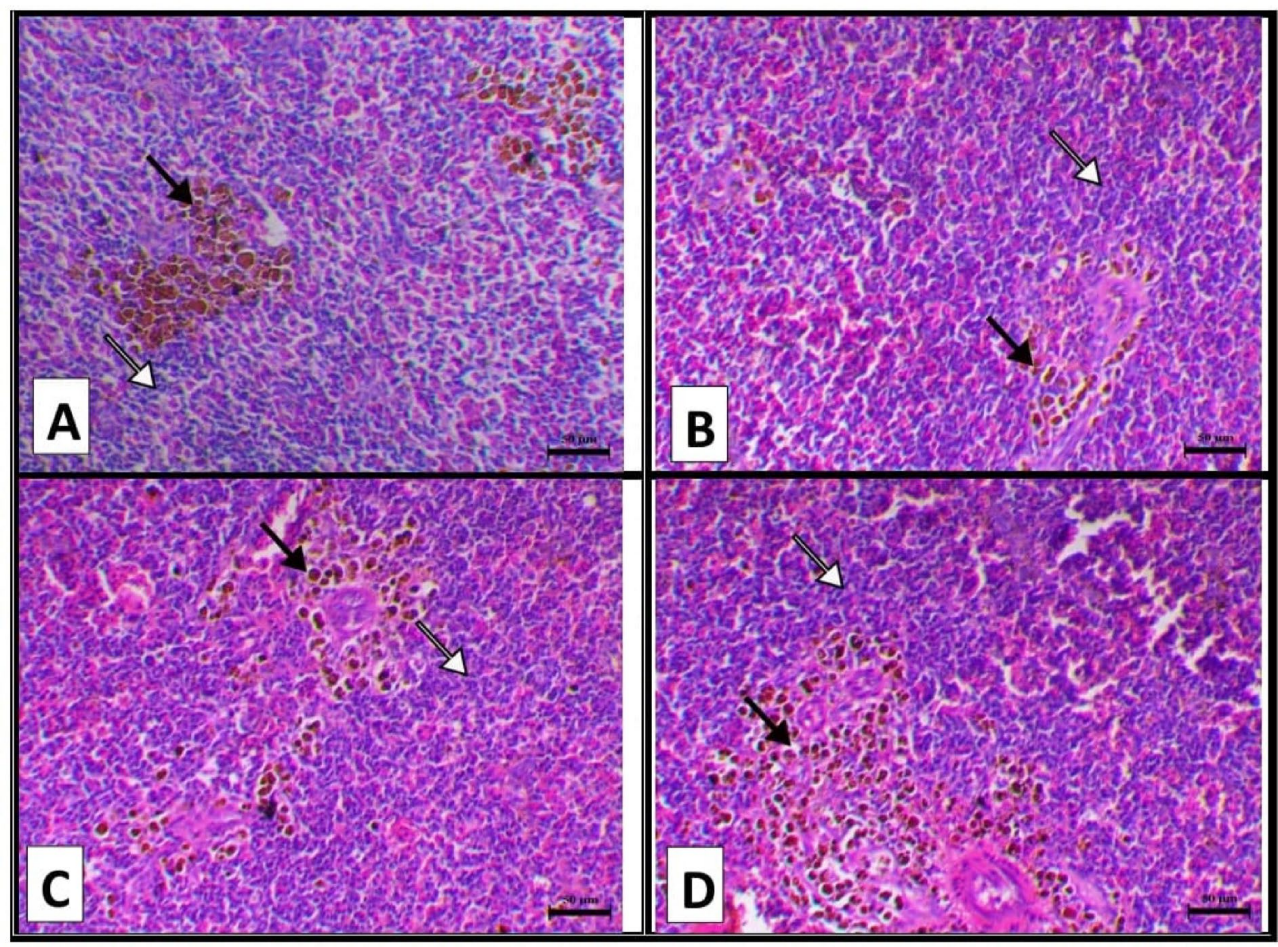
Showed the spleen of the different groups. (**A**) Spleen of fish supplemented with control diet showing normal lymphoid tissues including normal melanomacrophages cells (arrowhead). (**B**) Spleen of fish supplemented with biochar of Pomegranate peel 1gm/100 gm diet showing normal lymphoid tissues (white arrow) with few melanomacrophages cells (black arrow). (**C**) Spleen of fish supplemented with biochar of Pomegranate peel 3gm/100 gm diet showing normal lymphoid tissues (white arrow) with mild increase of melanomacrophages cells (black arrow). (**D**) Spleen of fish supplemented with biochar of Pomegranate peel 5gm/100 gm diet showing normal lymphoid tissues (white arrow) with marked increase of normal melanomacrophages cells (black arrow), H&E, X100, bar = 50 μm.

The results of the histology of the present study are shown in Figures 1, 2, and 3 and Table 7. Figure 1 shows the middle portion of the intestine of fish supplemented with a control diet showing normal villi lined with pseudostratified epithelium with goblet cells (A). The intestine (middle portion) of fish supplemented with 1% biochar of pomegranate peel showing a mild increase of intestinal villi length (B). The intestine (middle portion) of fish supplemented with 3% biochar of Pomegranate peel diet shows a mild increase of intestinal villi length (C). The intestine (middle portion) of fish supplemented with 5% biochar of Pomegranate peel diet shows a marked increase in villus length (D). Moreover, villi length and width and numbers of goblet cells significantly (*P* ≤ 0.05) increased in the biochar groups when compared with the control group. Meanwhile, intervilli space significantly (*P* ≤ 0.05) decreased in the Bp3% and Bp5% when compared with the Bp1% and control groups.

**Table 7:** Effect of different doses of Biochar of pomegranate Peel on chemical composition of nile tilapia body for 60 days.

Parameters Groups	Villi length (um)	Width Villi (um)	Inter villi space (um)	Goblet cells No/mm2
Control	386.80 ± 0.61 ^d^	74.37 ± 2.36 ^c^	64.47 ± 1.85 ^ab^	142 ± 3.21 ^c^
Bp1%	437.55 ± 3.33 ^c^	82.47 ± 3.35 ^b^	68.53 ± 0.93 ^a^	151 ± 2.00 ^c^
Bp3%	459.35 ± 3.52 ^b^	89.29 ± 0.63 ^ab^	57.11 ± 2.48 ^b^	176.67 ± 3.84 ^b^
Bp5%	797.88 ± 3.74^a^	90.52 ± 2.15 ^a^	40.92 ± 3.46 ^c^	417 ± 3.00 ^a^

Values are means ± standard error. Mean values with different letters at the same column significantly differ at (*P* ≤ 0.05). The absence of letter means that there were no significant differences between treatments.

Figure 2 shows the liver of fish supplemented with a control diet, showing normal hepatic and pancreatic portions with marked hepatic vacuolation (A). Liver of fish supplemented with biochar of Pomegranate peel 1% diet showing normal hepatic and pancreatic portions and decreased hepatic vacuolation (B). Liver of fish supplemented with 3% biochar of Pomegranate peel showing normal hepatic and pancreatic portions and decreased hepatic vacuolation (C). Liver of fish supplemented with 5% biochar of pomegranate peel showing normal hepatic and pancreatic portions and decreased hepatic vacuolation (D).

Figure 3 shows the spleen of fish supplemented with a control diet, showing normal lymphoid tissues, including normal melanomacrophages cells (A). Spleen of fish supplemented with 1% biochar of Pomegranate peel showing normal lymphoid tissues with few melanomacrophages cells (B). Spleen of fish supplemented with 3% biochar of pomegranate peel showing normal lymphoid tissues with a mild increase of melanomacrophages cells (C). Spleen of fish supplemented with 5% biochar of Pomegranate peel showing normal lymphoid tissues with a marked increase of normal melanomacrophages cells (D).

### Gene expression

**Fig. 4:**
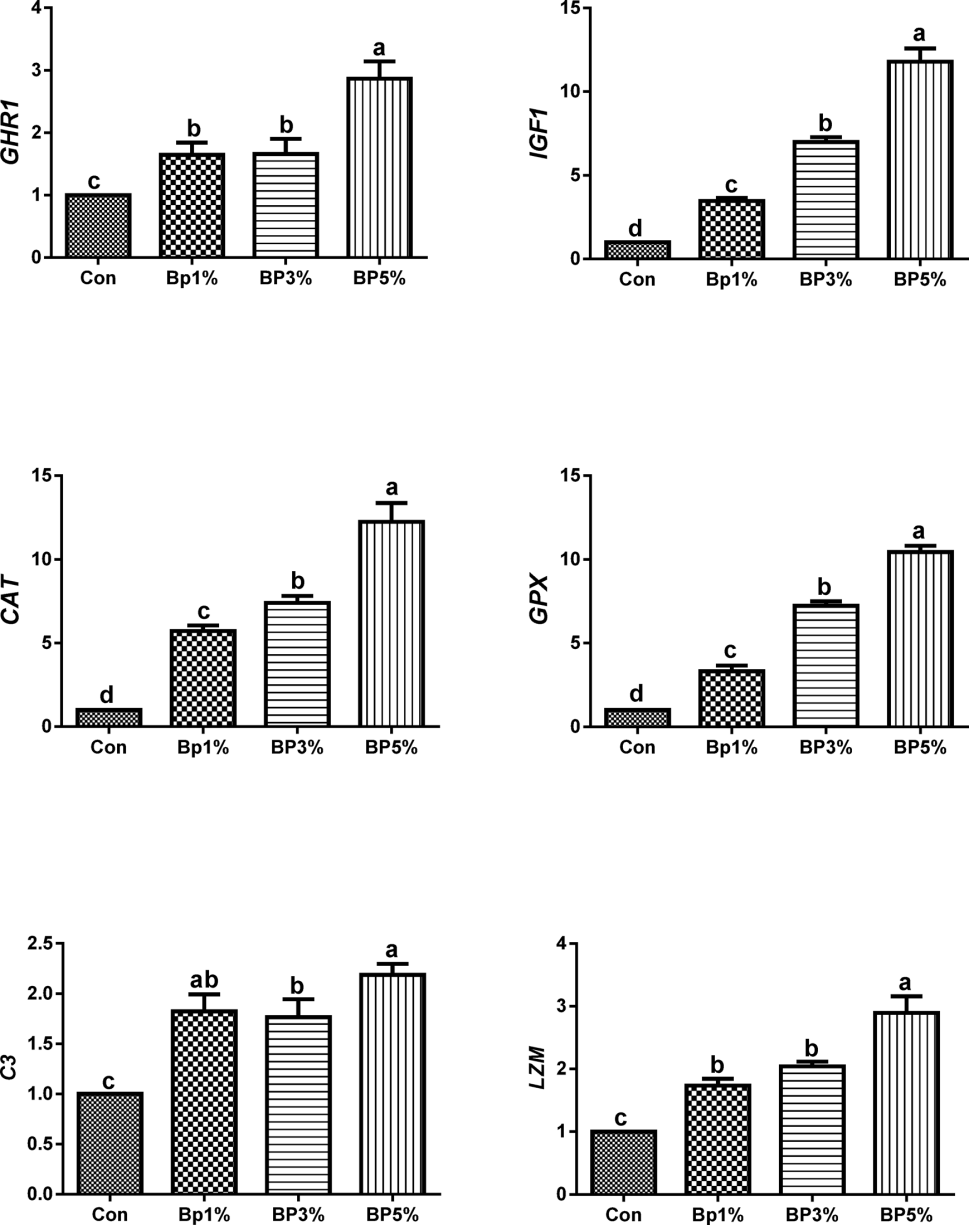
Shows the expression of the growth-related genes (*GHR1*, *IGF1*), antioxidant-related genes (*CAT*, *GPx*), and innate immunity-related genes (C3 and LZM) in the different groups treatments.

The expressions of the growth-related genes (*GHR1* and *IGF1*, antioxidant-related genes (*CAT* and *GPx*), innate immunity-related genes *C3 and LZM* are depicted in Fig. 4. The biochar groups (Bp1%, Bp3%, and Bp5%) exhibit overexpression of all measured genes.

### Economic indices

Table 8 represents the effect of different doses of biochar of pomegranate peel on economic indices. The lowest feed costs and total costs were recorded for the control group as compared to the pomegranate peel-supplemented groups (*P* ≤ 0.05). However, the highest TR, NP, RRI, and PI were observed for the groups fed diets containing 5% and 3% Bp (*P* ≤ 0.05), respectively, as compared to other groups without significant differences from each other. The lowest values for the abovementioned parameters were presented in the control and Bp1% groups without significant differences from each other as well.

**Table 8:** Effect of different doses of biochar of pomegranate peel on economic indices.

Parameters Groups	Feed cost (EGP)	TC (EGP)	TR (EGP)	NP (EGP)	RRI %	PI
Control	2.25 ± 0.02 ^d^	6.25 ± 0.02 ^d^	9.14 ± 0.25 ^b^	2.88 ± 0.27 ^c^	146.25 ± 4.42 ^c^	31.49 ± 2.13 ^b^
Bp1%	2.33 ± 0.01^c^	6.33 ± 0.01 ^c^	9.39 ± 0.30 ^b^	3.05 ± 0.32 ^bc^	148.26 ± 5.16 ^bc^	32.38 ± 2.44 ^b^
Bp3%	2.41 ± 0.01 ^b^	6.41 ± 0.01^b^	10.18 ± 0.12 ^a^	3.77 ± 0.11 ^ab^	158.86 ± 1.78 ^ab^	37.03 ± 0.70 ^ab^
Bp5%	2.47 ± 0.01^a^	6.47 ± 0.01^a^	10.55 ± 0.12 ^a^	4.07 ± 0.12 ^a^	163.04 ± 1.94 ^a^	38.65 ± 0.74 ^a^

Values are mean ± stander error. Mean values with different letters at the same column significantly differ at (*P* ≤ 0.05).

TC = Total cost; TR = Total Return; NP = Net Profit; RRI = Rate of return on Investement; PI = Profitability Index.

## Discussion

### Growth performance

Aquaculture is one of the food production industries with the quickest rate of growth in the world and is widely acknowledged as a crucial component of initiatives aimed at reducing poverty. Seafood and fish are among the most consumed foods in the world and are important sources of animal protein. After carp, Nile tilapia are the most commonly farmed freshwater species^2^. Plants and their extracts are frequently utilized as an unorthodox approach to immune stimulants, growth enhancers, and feed supplements^35^. Our research revealed that adding more pomegranate peel biochar to Nile tilapia diets can, up to a point, increase Nile tilapia growth and feed consumption. This is in line with the results of Amjad et al.^36^. Another study found that after 8 weeks of dietary activated charcoal (7.0 g/kg), Nile tilapia growth characteristics greatly improved^37^. Additionally, Najmudeen et al.^38^ demonstrated that when *Oreochromis mossambicus* was administered *Eichhornia crassipes* biochar at concentrations of 0.5% and 1%, it significantly increased in weight and length. Pomegranate consumption significantly improved the dietary value and growth of juvenile fish^20^. Moreover, Toutou et al.^9^ found that growth parameters of Nile tilapia may be enhanced by feeding them up to 5% per kilogram of pomegranate peel. Yousefi et al.^39^ observed that extract of pomegranate peel improves growth of common carp at 0.5% but retards growth when increasing concentration. Similar results were observed by Zakeri et al.^40^ with pomegranate seed oil in the common carp. However, Amjad et al.^36^ demonstrated that adding activated charcoal to aquafeeds can adsorb and eliminate gasses and impurities from the digestive system, improving nutrient absorption and utilization—a critical component of increased fish growth and feed efficiency. On contrary, Hussein et al.^41^ reported that pomegranate peel lowers body weight and feed intake and increases FCR of Nile tilapia. Additionally, Hamed and Abdel-Tawwab^42^ found a notable decline in the growth performance of *O. niloticus* at a dietary level of 3% and 5% per kg of pomegranate peel. Motlagh et al.^43^ found a significant decrease in the final weight and specific growth rate of *Carassius auratus* treated with 4% pomegranate peel extract. Meanwhile, Monir et al.^22^ found that when *O. niloticus* is given extract of pomegranate peel at rates of 0.3% and 0.5% per kg, its growth performance is unaffected. On the other hand, Avazeh et al.^44^ observed higher FCR in the rainbow trout. However, Sönmez et al.^45^ found that extract of pomegranate peel lowers final body weight and FCR of rainbow trout. Moreover, Fayed et al.^46^ discovered that rabbit bucks fed diets supplemented with pomegranate peel at doses of 5, 10, and 15 g/kg had lower final weights and growth rates than the control group. Also, when fed large amounts of pomegranate meal, Nile tilapia grew more slowly and consumed less feed^19^. Tawfik et al.^47^ reported that 20% orange pulp negatively affected the growth performance of Nile tilapia. However, according to Van et al.^48^, giving goats active charcoal supplements greatly improved their apparent dry matter and protein digestibility. Additionally, Pirarat et al.^49^ found that the processes of fish digestion and absorption may benefit from dietary active charcoal supplementation. Reda et al.^50^ found that olive stone biochar enhances the growth of *O. niloticus*, which is exposed to inorganic mercury. Nonetheless, results of the gene expression of the growth-related genes validate the growth performance results.

### Proximate analysis of the fish body

The results of the chemical composition come in line with Nogales-Mérida et al.^51^, who reported that feeding *Diplodus puntazzo* citrus pulp silage, which has a high pectin content, enhances moisture and lowers crude lipid. On the other hand, the fat% of the *O. niloticus* body was verified by Sinha et al.^52^, who found that feeding rainbow trout with 10% guar gum resulted in a significant drop in fat content. Similar results regarding fat% and ash% were obtained by Tawfik et al.^47^ with Nile tilapia fed orange pulp. Additionally, Hussein et al.^41^ noted that the amount of ash and protein in the Nile tilapia body varies negligibly between treatments with different doses of pomegranate peel. It is important to note that the findings of this study showed that increasing the amount of dietary biochar in *O. niloticus* decreased the amount of carcass protein. On the contrary, Abdel-Tawwab et al.^37^ and Boonanuntanasarn et al.^53^, Michael et al.^54^, and Thu et al.^55^ observed that increasing the amount of dietary charcoal in *O. niloticus*, red tilapia, and Japanese flounder, respectively, increases the amount of carcass protein. However, Al-Sokary et al.^56^ recommended using pomegranate peel extract to increase the overall quality and storage life of fish. On the contrary, Tawfik et al.^47^ reported that 20% orange pulp increased the crude protein of the Nile tilapia body.

### Biochemical parameters

Serum biochemical characteristics can serve as a gauge of overall health^57^. Thus, the biochemical parameters were measured to assess the effect of biochar on fish health. However, the findings of the serum biochemistry are confirmed by the findings of El- Badrey et al.^19^, Munir et al.^58^, and El-Sayed et al.^59^, who reported that a meal rich in pomegranates lowers the blood levels of urea, creatinine, cholesterol, aminotransferase, and aspartate aminotransferase in Nile tilapia. At 0.5% and 1% pomegranate seed oil, fish’s ALP, AST, and ALT concentrations drop^10^. According to Mabe et al.^60^, adding 4% bamboo charcoal to the diet of common carp enhanced blood biochemical indicators, including ALT, AST, and total protein. Moreover, Abdel-Tawwab et al.^37^ found that adding 10–20 g of active charcoal kg − 1 to the diet of Nile tilapia improved fish performance overall and lessened the adverse effects of a synthetic mixture of lead, cadmium, copper, and zinc on ALT, AST, urea, and creatinine. On the contrary, Tawfik et al.^47^ reported that orange pulp increased activity of liver enzymes. Also, Van Doan et al.^61^ reported that serum peroxidase was considerably elevated when orange peel pectin was added to the diet.

### Antioxidant

Fish raised under intensive culture with limited resources experience stressful situations^62^. Stress has a detrimental effect on fish, increasing their metabolism and generating reactive oxygen species^36^. Polyphenols present in the pomegranate peel keep the immune system in balance and have anti-inflammatory and antioxidant effects^63^. The findings of the antioxidant are consistent with the results of Sarfraz et al.^20^, who reported that pomegranate consumption significantly improved antioxidant activity of juvenile fish. Moreover, our results are consistent with earlier studies that looked at the impact of dietary orange peel at dosages of 0, 1, 3, and/or 5 g/kg diet on sea bream diets^64^. Abd El-hameed et al.^65^ observed that charcoal lower the level of MDA in *O. niloticus*, which is subjected to sub-lethal imidacloprid toxicity. Additionally, Vicente et al.^66^ investigated the effects of heat and dissolved oxygen on TAC and CAT in *O. niloticus* under stress at varying doses of orange peelings in their diets (0, 2, 4, 6, and/or 8 g/kg). Giri et al.^67^ found that the antioxidant activity of *Labeo rohita* was enhanced by dietary banana peel flour. Protochatechuic acid, gallic acid, pyrogallol, coumaric acid, catachine, rosmarinic naringeen, myrcetin acid, scoplatin, and hisperdin are among the many antioxidant substances found in pomegranates^42^. Hamed and Abdel-Tawwab^42^ and Munir et al.^58^ reported that the activities of SOD and CAT in fish are raised with dietary pomegranate. Moreover, results of Sönmez et al.^45^ indicate that the activity of SOD is increased when the fish are fed pomegranate at a rate of 0.025% and 0.05% per kilogram of feed. A fish diet that contains 1% extracts of three different fruits—pomegranate, banana, and orange—may raise the amount of albumin, globulin, and total protein in the fish’s blood^68^. Moreover, El-Bouhy et al.^69^ found that pomegranate peel decreased subchronic toxicity of mercury in *Clarias gariepinus* by increasing antioxidant parameters. Additionally, Reda et al.^50^ observed that olive stone biochar enhances the antioxidant of *O. niloticus*, which is exposed to inorganic mercury. Nonetheless, results of the gene expression of the antioxidant-related genes validate the antioxidant results.

### Histology

The study’s findings demonstrated that feeding *O. niloticus* a pomegranate peel biochar diet can reduce tissue damage and increase their resilience to oxidative stress. The presence of antioxidants in pomegranate peel biochar may be the cause of this. Our results are consistent with earlier studies that looked at the impact of dietary orange peel at dosages of 0, 1, 3, and/or 5 g/kg diet on sea bream diets^64^. Hussein et al.^41^ observed increased goblet cells with increased pomegranate peel in the diet of Nile tilapia. According to Thu et al.^55^, activated charcoal may also have the ability to condition intestinal cell membranes, lower surface tension by removing gases, toxins, or other harmful substances from the intestine, and enhance nutrient absorption and utilization across cell membranes. Pirarat et al.^49^ reported that activated charcoal improved intestinal villi length of *O. niloticus* and consequently improved growth. Michael et al.^54^ found that charcoal improve the growth of red tilapia by improving intestinal villi length and enterocytes.

### Gene expression

The *GH* and *IGF1* genes regulate fish development and cell activity through a variety of signaling pathways^70^. The gene expression findings in the *O. niloticus’s* liver tissue corroborated the performance finding. According to a number of studies, adding fruit peel to feeds of fish in the right amounts can improve fish growth, immunological response, and general health^71,72^. Additionally, dragon fruit peel increased expression of antioxidant and immune-related gene expressions^72^. Tawfik et al.^47^ reported that orange pulp increased *RNA* levels of *GHR* in liver tissue of *O. niloticus*. It has long been understood that complement is a component of the innate immune system that protects the host from the invasion of foreign infections. One of the main components of the complement system is *C3*^73^. On the other hand, Harikrishnan et al.^74^ and Harikrishnan et al.^75^ adding dried lemon peel and grape pomace flour to the diet markedly increased *Labeo rohita* antioxidant gene expressions. *GSR* was found to be up-regulated in *O. niloticus* that were given 20 g/kg of powdered longan seed^76^. Additionally, Qiao et al.^77^ and Van Doan et al.^78^ reported that Poly-β-hydroxybutyrate and pineapple peel powder increased immune-related gene expression in *gibel carp* and *O. niloticus*, respectively. Outama et al.^79^ reported that although growth performance and antioxidant gene expressions were not considerably improved, immune response and immune-related gene expressions were improved. Mohammady et al.^80^ found that GPX gene expression is markedly reduced by a dietary phytogenic mixture derived from garlic, onion, and lemon. Divergent experimental conditions, such as fish species, culture duration, and water quality, may be the cause of these discrepancies.

### Economic analysis

The findings of the current study further revealed that fish receiving diets supplemented with pomegranate peel biochar exhibited significantly enhanced total return, net profit, rate of return on investment, and profitability index. This is in agreement with Sulaiman et al.^81^ and Kawata et al.^82^, who suggested that the incorporation of fruit waste in aquaculture feed can improve the profitability of tilapia farming. These results are attributed to the higher growth rate and weight gain achieved by the addition of BP, as demonstrated by Amjad et al.^36^ and Toutou et al.^9^, as well as better FCR and feed efficiency compared to the control group Sulaiman et al.^83^. Therefore, pomegranate peel biochar could be a cost-effective and economically viable additive in the diet of Nile tilapia.

## Conclusion

The current study unequivocally shown the significance of feeding the fish pomegranate peel biochar under the present experimental setting. The results indicated that in addition to improving growth parameters and lowering MDA, the addition of pomegranate peel biochar increased TAC and CAT in *O. niloticus* fry. These enhanced the fish’s overall health because of the natural antioxidants found in the pomegranate peel biochar. Additionally, biochar overexpressed the growth, antioxidant, and immune-related gene in the liver tissue with higher villi length and an increased number of goblet cells without any pathological lesions. Moreover, the economic parameters of Nile Tilapia farming greatly improved by the incorporation of pomegranate peel biochar. Therefore, it is beneficial to focus on using pomegranate peel biochar as a growth promotor in farmed fish.

## Data Availability

The datasets used and/or analyzed during the current study available from the corresponding author on reasonable request.

## Declarations

### Guidelines

All methods were carried out in accordance with relevant guidelines and regulations.

### ARRIVE guidelines

The authors confirm that the study was carried out in compliance with the ARRIVE guidelines.
